# TYPOLOGIES OF PARENT-CHILD RELATIONSHIPS AND ASSOCIATED HEALTH OUTCOMES: A CROSS-CULTURAL COMPARISON

**DOI:** 10.1093/geroni/igac059.911

**Published:** 2022-12-20

**Authors:** Dexia Kong, Peiyi Lu, Merril Silverstein

**Affiliations:** The Chinese University of Hong Kong, Hong Kong, Hong Kong; Columbia University, New York, New York, United States; Syracuse University, Syracuse, New York, United States

## Abstract

**Background:**

This study investigates types of parent-child relationships and associated health outcomes among older adults of U.S. and China. Method: Cross-sectional data from Health and Retirement Study in the U.S. and Health and Retirement Longitudinal Study in China were used (N_(U.S.)=3918, N_China=4058). Relationship indicators included co-residence, living nearby, having weekly contacts, receiving assistance with daily activities, providing grandchild care, and having financial transfer from/to children. Latent class and regression analyses were conducted.

**Results:**

Four classes were identified for older Americans, including (1) distant and uninvolved (6.58%); (2) geographically proximate with frequent contacts and downward support (47.04%); (3) co-resident with frequent contacts and upward support (13.1%); and (4) geographically proximate with frequent contacts (33.28%). By contrast, three classes were identified among older Chinese, including (1) co-resident with frequent contacts and upward support (37.46%); (2) coresident/interdependent (25.65%); (3) geographically proximate with frequent contacts and upward financial support (36.89%). For both countries, providing downward support was associated with fewer functional limitations and better cognitive function. Receiving instrumental support from children was associated with more depressive symptoms and functional limitations, and poorer cognitive function among Chinese older adults only.

**Conclusions:**

Cultural contrasts were evident in parent-adult child relationship types and their associations with health outcomes. Overall, child-parent relationships in China tend to be more tight-knit than that of the U.S. Receiving financial support from children and co-residence are unique features of child-parent relationships in China. Cultural differences in child-parent relationships call for culturally-relevant strategies to address needs of older adults from various cultures.

